# Vacation Visits

**Published:** 1884-08

**Authors:** 


### VACATION VISITS.

ONE of the principal advantages of vacation outings is that of change—change of companionship, change of scene, change of food, and change of air. To some the scenes and associations and breathings of the seaside are a grateful change. To others the dim forests, the balsamic air of piny woods, the breezy perches on the mountain top, are the necessary changes to give a new impetus to the sluggish blood and new ideas to the tired brain. In either case a sense of rest and freedom from care must accompany the change of locality, or all the benefit of the effort is lost. The "shop" must be left behind.

But the air and sun are the great curatives. The seaside goers imagine that the surf bath is the reason and secret of restored energy; but they give too little credit to the open-eyed sun and free blowing air of the seashore. An air bath and sun bath have as much to do with renovation of jaded human frames as the direct contact of salt water. The Philadelphia *Ledger* says that "the tonic influence of the salt air is, at least, equal to that of the bath, and it may be superior. At the seashore a large proportion of the daily life of the visitor is out door life, as contrasted with the indoor habit of many (and, indeed, most people) during the rest of the year. There is that health giving change to begin with. The visitor has more fresh air. Then, as to the air itself. First, it is free from the many impurities that more or less pervade the atmopheres of large and densely compacted cities; the products of combustion thrown out from hundreds of thousands of chimneys; the exhalations from a crowded population; the gases from factories, laboratories, culverts, closets, and various other sources of contamination that need not be recited here. Second, the shore air, almost exclusively from the sea, bears wholesome, natural elements with it, so subtle and penetrating that their precise individual influence cannot always be traced, but we know what the effects are in their health renewing combination."

